# Association of IL28B SNP With Progression of Egyptian HCV Genotype 4 Patients to End Stage Liver Disease

**DOI:** 10.5812/hepatmon.835

**Published:** 2012-04-30

**Authors:** Mostafa K. El-Awady, Lotiaf Mostafa, Ashraf A. Tabll, Tawfeek H. Abdelhafez, Noha G. Bader El Din, Naglaa Zayed, Reem El Shenawy, Yasmin El Abd, Reham M. Hasan, Hosam Zaghlol, Hesham El Khayat, Ashraf O. Abdel Aziz

**Affiliations:** 1 Department of Microbial Biotechnology, National Research Center, Giza, Egypt; 2 Ahmed Maher Educational Hospital, Cairo, Egypt; 3 Department of Tropical Medicine and Hepatology Kasr El Aini Cairo University, Cairo, Egypt; 4 Faculty of Medicine, Mansoura University, Mansoura, Egypt; 5 Theodor Bilharz Research Institut, Cairo, Egypt

**Keywords:** Hepatitis C, Interleukin 28B, Polymorphism, Genetic, Liver Cirrhosis, Carcinoma, Hepatocellular

## Abstract

**Background:**

IL28B single nucleotide polymorphisms (SNPs) play important roles in the management of hepatitis C virus (HCV) infections and are strongly associated with spontaneous and treatment-induced HCV clearance.

**Objectives:**

In the present study, the association between IL28B variants and the progression of HCV infection in Egyptian patients infected with type 4a virus will be examined.

**Patients and Methods:**

Frequencies of the protective genotype C/C of SNP, rs12979860 were determined in healthy subjects, spontaneous resolvers, and chronic HCV type 4 patients with low F scores and in patients with end stage liver disease (ESLD). This study included a total of 404 subjects. Patients infected with HCV type 4a (n = 304) were divided into; chronic hepatitis C (CHC) with low F scores (CHC, n = 110), end stage liver disease (n = 110), liver cirrhosis (LC) (n = 35) and hepatocellular carcinoma (HCC) patients (n = 75), spontaneous resolvers of HCV infection (n = 84) were also included. A healthy group representing the Egyptian population (n = 100) was also included in the genotyping of IL28B. The later was typed via a polymerase chain reaction based restriction fragment length polymorphism (PCR-RFLP) assay analysis on purified genomic DNA extracted from all individuals.

**Results:**

A significant increase (P < 0.0005) was observed in frequencies of IL-28B rs12979860 C/C genotypes in the healthy population, than in the CHC, LC and HCC groups (C/C = 48%, 13%, 0%.and 0% respectively). On the other hand the C/C genotype was significantly higher (P < 0.0005) in spontaneous resolvers than in healthy subjects. A comparable significant increase in the frequency of C/T allele accompanied by mild elevation of T/T allele frequency, were detected along the progression towards ESLD.

**Conclusions:**

Genotype C/C is associated with viral clearance during acute infection. The sharp decline in the C/C genotype from healthy to CHC subjects and the total absence of the C/C genotype in ESLD suggests a central role of this genotype against HCV disease progression.

## 1. Background

Several immunological factors have been implicated in determining disease outcomes in hepatitis C virus (HCV) infections [1][2] Approximately 30% of individuals clear the infection naturally, whereas the remaining 70% develop chronic disease that may result in liver cirrhosis (LC) and/or hepatocellular carcinoma (HCC) [3][4]. Therefore, identification of the factors involved in persistent HCV infections may lead to the development of effective prognostic tests and hence improved treatment management or to the development of novel antiviral agents. Although the role of the adaptive immune response has been well documented [2], other evidence [5][6] supports a role for the innate immune system in regulating disease progression in HCV infection. More recent evidence to support a role for the innate immune system in HCV outcomes, has come from a series of studies on SNPs in the IL28B gene region which predicts spontaneous and type 1 IFN induced clearance of HCV infections. Multiple genome-wide association studies (GWAS) have identified single nucleotide polymorphisms (SNPs) near the IL28B gene (encoding IFN-λ3) to be strongly associated with spontaneous and treatment-induced clearance of HCV infections [7]. One of these SNPs, rs12979860 [8] was pivotal in predicting the resolution of HCV infections [9][10]. The SNP rs12979860 is found ~ 3 kb upstream from the IL28B gene. Little is known about the IFN λ family, but evidence is mounting to support a role for them in the immune response to viral infections [11][12]. Therefore, associations made between IL28B variants and HCV clearance in large-scale genetic studies provides an exciting mechanistic link between innate immunity and viral clearance. Chronic HCV patients (CHC) can be roughly categorized into patients with a very slow disease progression and patients with rapid progression into LC and HCC. The factors controlling the pathobiology of HCV disease are either viral or host related. No apparent differences between the pathobiology of HCV genotypes was reported until Mihm et al. [13] identified a relationship between hepatic steatosis and HCV genotype 3 infections. This relationship was subsequently confirmed by comparing patients infected with genotype 3 and those infected with other genotypes [14][15][16]. However, Genotype 4a represents more than 93 % of chronic HCV patients in Egypt [17][18].

## 2. Objectives

The aim of the present study is to examine the association between IL28B variants and the progression of HCV infection in Egyptian patients infected with type 4a virus. The incidence of the protective genotype C/C of SNP, rs12979860 located ~ 3 Kb upstream of the IL 28B gene was screened in healthy Egyptian subjects, chronically infected HCV patients with low F scores, spontaneous resolvers and in HCV type 4a patients with LC or HCC.

## 3. Patients and Methods

Serum α fetoprotein levels were routinely tested in all patients with decompensated cirrhosis. AFP levels were generally elevated in cases that were proved to be HCC, however, the levels ranged from 15 to 650 ng/ml. The diagnosis of HCC was confirmed by a 4 phase multidetector computed tomography (CT) scan or dynamic contrast enhanced magnetic resonance imaging (MRI).

### 3.1. Patients

a) Healthy subjects (n = 100, 50 males and 50 females) with no history of liver infection, long term drug use, autoimmune hepatitis or alcohol consumption were selected. Age of the healthy subjects ranged from 18-64 years with a median value of 41 years.

b) Spontaneous resolvers (84 subjects; 53 males and 31 females) who tested positive for IgG anti HCV Abs (3rd generation) and negative for HCV RNA, were enrolled in the study.

c) Chronic HCV patients (n = 110, 85 males and 25 females, age 22-65 years with a median value of 43.5 years) with limited pathogenesis, i.e. mild, elevated or normal hepatic enzymes. All chronic HCV patients had mild liver disease F0-F2, HAI1 - HAI6 according to the METAVIR histological liver biopsy grading. Patients with diabetes, thyroid dysfunction, coinfection with HIV or hepatic pathogens such as; HBV DNA, CMV DNA, and Anti schistosomiasis Abs were excluded.

d) Decompensated cirrhosis patients (n = 35, 22 males and 13 females, aged 31-62 years with a median value of 46.5 years) with end stage live disease (ESLD) due to chronic HCV type 4a infection without the involvement of other hepatic factors such as; auto immune hepatitis, hepatitis B virus (HBV), alcohol hepatotoxicity, or a history of aflatoxin hepatotoxicity. Some patients were suffering from ascites, or esophageal bleeding with no hepatic focal lesions.

e) Hepatocellular carcinoma patients (n = 75, 57 males and 18 females, aged 34-67 years with a median value of 50.5 years) with multiple lesions and a history of liver cirrhosis on top of a chronic HCV infection. None of the patients had any other hepatopathogenic etiology. The diagnosis of HCC was made after reviewing images generated with several imaging modalities. Nodules larger than 1 cm found in the ultrasound screening of a cirrhotic liver were investigated further with either 4phase multidetector computed tomography (CT) scan or dynamic contrast enhanced magnetic resonance imaging (MRI). If the appearances were typical of HCC, namely, hypervascular in the arterial phase with washout in the portal venous or delayed phase, the lesion was diagnosed as HCC. If the findings were not characteristic or the vascular profile was not typical, a second contrast enhanced study with the other imaging modality was performed or the lesion was biopsied. All patients were confirmed not to have other cancers in an initial screening examination.

### 3.2. Detection of IL-28B rs12979860 C/T Polymorphism

#### 3.2.1. Samples

Peripheral blood on EDTA was withdrawn from all subjects and genomic DNA was extracted using genomic DNA extraction kits (Qiagen, Milan Italy). Plasma were separated before DNA extraction and utilized for testing; HBV sAg, anti schistosomiasis Abs and autoimmune Abs (ANA, AMA, LKA). Since CMV reactivation was recently shown by our laboratory to interfere with several innate immunity pathways and subsequently poor drug induced viral clearance rates [19], CMV DNA was tested in extracted DNA from all subjects to exclude subjects with detectable CMV DNA from the study.

#### 3.2.2. Genotyping for the IL-28B rs12979860 C/T Polymorphism

**Figure 1:**
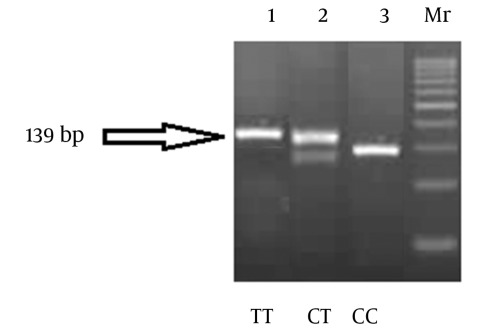
PCR- RFLP Analysis of rs 12979860 IL28B SNP Using an Assay Described in Materials and Methods. (Three different healthy individuals were typed and the products were run on agarose gel electrophoresis (4%) to resolve restricted fragments after bstU1 digestion. 139 bp indicates TT genotype, 109 bp indicates CC genotype, 139 + 109 bp indicates CT genotype)

Genotyping for the IL-28B rs12979860 C/T polymorphism was performed by a polymerase chain reaction based restriction fragment length polymorphism (PCR-RFLP) assay. Genomic DNA was extracted from whole blood samples by means of the QIAamp DNA blood mini kit (Qiagen, Milan, Italy) according to the manufacturer’s instructions. A 139 base pair (bp) product was obtained with the forward primer 5`- CCAGGGCCCCTAACCTCTGCA - 3` and the reverse primer 5`- GGGAGCGCGGAGTGCAATTCA - 3`, newly designed with the aid of the NCBI Primer-Blast Tool (http://www.ncbi.nlm.nih.gov/tools/primer-blast/). PCR amplification was carried out in a total volume of 10 ul containing 10 mM Tris–HCl (pH 8.3), 50 mM KCl, Tween-20 0.01%, 0.2 mM deoxyribonucleotides, 2–4 pmol of each primer, 2.0 mM MgCl2, 0.5 units hotstart Taq DNA polymerase (Right Taq, Euroclone, Milan, Italy). Samples containing 10 ng of genomic DNA were subjected to 35 cycles of denaturation (at 95 0C for 60 s), annealing (at 64 0C for 60 s), and elongation (at 72 0C for 60 s). Ten ul of the amplicons were digested with 1 unit of the BstU-I restriction endonuclease (New England Biolabs, Hitchin, UK) in a total volume of 20 ul at 37°C overnight. The fragments were resolved by electrophoresis on 4% agarose gel after staining with ethidium bromide. A band of 139 bp indicates the T/T genotype, 109 bp + 30 bp (usually invisible on the gel) indicates the C/C genotype, and 139 bp + 109 bp+ 30 bp (invisible) indicates the C/T genotype (Figure 1).

### 3.3. Statistical Analysis

All statistical analyses were performed using the SPSS 9.0 statistical software program. A statistical significance was considered when P ≤ 0.05. IL28B SNP genotype analysis was carried out according to Santiago Rodriguez, Tom R. Gaunt and Ian N. M. Day, with the Hardy-Weinberg Equilibrium Testing of Biological Ascertainment for Mendelian Randomization Studies. American Journal of Epidemiology Advance Access published on January 6, 2009, DOI 10.1093/aje/kwn359. http://www.oege.org/software/hwe-mr-calc.shtml

## 4. Results

### 4.1. Genotyping of IL28B in Healthy Subjects

**Figure 2:**
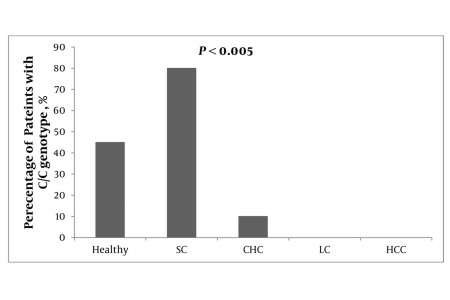
Frequency of rs12979860 C/C in all 320 Study Participants. Healthy population (n = 100), chronic hepatitis C (CHC; n = 110), liver cirrhosis (LC; n = 35) and hepatocellular carcinoma (HCC; n = 75)

To type the rs 12978860 SNP among the healthy Egyptian population, the C/T polymorphism was determined in a sample of 100 subjects that had neither a history of viral hepatitis infection nor other chronic inflammatory diseases that may reflect poor innate subject immunity. As demonstrated in Figure 2 the protective genotype C/C constituted 48% of the studied sample, while the T/T genotype constituted 14 % and the heterozygous genotype C/T 38%. The genotype analysis of the SNP revealed that the protective C allele was significantly more prevalent than the T allele (67% C vs. 33% T) in a total of 200 alleles (100 subjects) tested in the present study.

### 4.2. IL 28 C/C Frequency in Spontaneous Resolvers of HCV Infection

To test the hypothesis, do patients who spontaneously clear HCV infection during or shortly following the acute phase bear the protective C/C genotype more frequently than other groups studied, i.e. healthy, CHC, LC and HCC patients, we typed the IL28 B SNP (rs 12979860) in 84 subjects with a spontaneous clearance of their HCV infection, i.e. detectable HCV Ab and negative HCV RNA in their sera. The results clearly indicated the protective role of the C/C genotype in patients who spontaneously cleared the virus, where C/C represented 86% of spontaneous resolvers vs. 48, 13, 0 and 0% in healthy subjects, CHC, LC and HCC patients respectively (Figure 2).

### 4.3. IL28B Variants in Chronic HCV Patients

**Figure 3:**
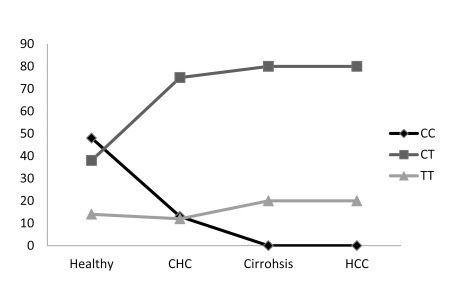
Frequencies of rs12979860 C/C, C/T, T/T. Il28B SNP was typed among healthy, chronic hepatitis C (CHC), cirrhotic and hepatocellular carcinoma (HCC) patients. The frequencies of IL28B variants were represented as % of total number of subjects in each group

The results of IL28B typing in 110 chronic HCV patients with a low F score (F0-F2) regardless of their response to pegylated IFN + RBV combined therapy are depicted in Table 1 and Figure 2 and Figure 3. A sharp decrease in the incidence of the protective C/C genotype and C allele compared with the typing data of healthy subjects was observed, 13 % C/C in CHC vs. 48% C/C in healthy subjects and 50% C allele in CHC vs. 67% C allele in healthy subjects. Such a decline in the C/C genotype was not reflected in a comparable increase in the T/T genotype; however a two fold increase (75% vs. 38%) in the heterozygous genotype C/T was observed in the CHC group compared with the healthy subjects.

**Table 1:** Distribution of IL28B rs12979860 SNP Alleles and C: T Ratios a

	**CC, No. (%)**	**CT, NO. (%)**	**TT, No. (%)**	**C:T ratio, %**
Healthy (n = 100)	48 (48)	38 (38)	14 (14)	67 : 33
SL (n = 110).	72 (86)	6 (7)	6 (7)	89.5 : 10.5
CHC (n = 110)	14 (13)	83 (75)	13 (12)	50 : 50
ESLD (n = 110 )	0.0 (0.0)	88 (80)	30 (20)	40 : 60

^a^ IL28B single nucleotide polymorphisms (SNPs) was typed via a polymerase chain reaction based restriction fragment length polymorphism (PCR-RFLP) -BstU1 analysis on purified genomic DNA extracted from all individuals

### 4.4. Typing of IL28B in HCV Patients With End Stage Liver Disease (ESLD)

The results of IL28B typing in ESLD (LC and HCC) are depicted in Table 2 and Figure 2 and Figure 3. The most interesting results of the current study are the entire absence of the protective C/C genotype in HCV patients with ESLD. The IL28B typing profile in this patient cohort displayed a clear inversion of C to T ratios to become 40%:60% instead of 67%:33% found in healthy subjects and 50%:50% in CHC patients. It is clear from the data presented in Figure 3 that ratios of both the C/C and C/T genotypes in different cohorts were exchanged as the progression of the HCV disease takes place, i.e. the C/C genotype continues to decline till it reaches its lowest value in the ESLD patients, while the C/T genotype continues to increase, progressing till it reaches its peaks in the ESLD group with no significant change in the incidence of the T/T genotype. Of the four patient groups, IL28B rs 12979860 genotypes were observed to have a statistically significant association with the outcomes of HCV infections (Table 2).

**Table 2:** Chi-squared and Hardy-Weinberg Equilibrium Testing of IL28B Genotype Distribution in Different Outcomes of HCV Infection, i.e. Spontaneous Clearance, Persistent Infection and End Stage Liver Disease as Compared With Healthy Controls

**Patient group**	**X^2^^a^**	**Genotype**	**Expected**	**Observed**	**C Allele Frequency**	**T Allele Frequency**
Control	1.98	CC	44.89	48	0.67	0.33
		CT	44.22	38		
		TT	10.89	14		
Spontaneous Clearance	39.38	CC	80.1	86	0.9	0.11
		CT	18.8	7		
		TT	1.1	7		
Chronic HCV	25.02	CC	25.5	13	0.51	0.5
		CT	50	75		
		TT	24.5	12		
ESLD	44.44	CC	16	0	0.4	0.6
		CT	48	80		
		TT	36	20		

^a^ Chi-squared, the X(2) value indicates the difference between expected and observed values for genotype counts

## 5. Discussion

As a result of the powerful genetic approach genomewide association study (GWAS), hundreds of thousands of SNPs spanning all chromosomes were mapped to distinct phenotypes or traits [20]. Several studies have linked the IL28B SNP rs 12979860 to treatment induced as well as spontaneous clearance of HCV infections. Members of the interferon lambda group such as IL28B and IL 29, both use the JAK-STAT signal cascade in a similar fashion to interferon-α and induce interferon stimulated genes, ISGs [21][22]. The later have been shown to play an important role in HCV treatment outcomes and spontaneous clearance of the virus [22]. Whether IL 28B plays a role in supporting innate immunity against severe pathogenesis, either via control of viral replication or through independent pathways to halt the disease progression is not yet clear. The current study was conducted to examine whether the C/C genotype of rs 12979860 SNP, located in the promoter region ~ 3 kb upstream of the IL28B gene, a member of the IFN λ innate control gene family, is significantly reduced in the Egyptian population infected with HCV type 4a and suffering from advanced liver pathobiology. We hypothesized that genotype C/C of the IL28B is associated with an inherited tendency towards protection against the progressive development of ESLD (LC and HCC). Spontaneous clearance of the HCV occurs in only 15–50% of all HCV infected individuals, while the majority of patients develop a chronic infection. Thomas et al. [8] found that IL28B rs12979860 is strongly associated with the chance to clear HCV spontaneously in populations of African or European ancestry, with an approximately three times higher clearance rate in individuals with the rs12979860 genotype C/C versus C/T, T/T. In the present study IL28B SNP was carefully typed into four distinct cohorts; namely healthy Egyptian subjects, spontaneous resolvers with detectable HCV Abs and undetectable HCV RNA, chronic HCV type 4a patients with F0-F2 fibrosis and a fourth group of ESLD due to HCV type 4a infection (decompensated LC and HCC). Genotyping of IL28B rs 12979860 in a sample of the normal Egyptian population (representing major ethnic backgrounds; Caucasian whites, Nubians, Bedouins and Egyptian Copts) revealed that the C/C genotype comprises the highest incidence; (48%) compared with the other two genotypes T/T (14%) and C/T (38%). The C:T allele ratio in the studied healthy subjects is 67:33, a figure that puts Egypt in the middle between the highest ratios found in populations from Asian and the lowest ratio in populations with African ancestry. Several global studies revealed that the C/C genotype is the major player in drug induced viral clearance, while both the C/T and T/T genotypes have a poor association with clearance rates [23][24][25]. The frequency distribution of rs 12979860 SNP with C/C approximating 50% is closest to the Turkish population among the frequencies reported in diverse populations worldwide. This incidence rate is supported historically in light of the long (400 year) Ottoman occupation in Egypt and the high incidence of interbreeding between the two populations. These data indicate that unlike populations with African ancestry, there are no genetic barriers among the Egyptian HCV patients prohibiting the control of HCV infections.

IL-28B rs12979860 C/T polymorphism appeared to predict the rate of spontaneous clearance of HCV infections, which could be observed in the 53.0% of patients with a C/C genotype vs. 23.4% of patients with a T/T genotype [8]. In the present study, the observed decline in C/C frequency among chronic HCV patients vs. healthy subjects (13% vs. 48%) highlights the protective role of IL28B C/C against progression to chronic infection via spontaneous clearance of the virus during the acute phase. A comparable increase in the T/T genotype was not observed in CHC patients indicating other factors are involved in viral persistence during progression to a chronic state. The current data shows that the decline observed in the C/C genotype was replaced by a corresponding increase in the frequency of the heterozygous C/T genotype, thus suggesting that phenotypic expression of the T allele is dominant over the C allele in heterozygous patients. The simultaneous decline of the C/C genotype frequency to one quarter of the value in healthy subjects and doubling of the C/T genotype in CHC patients have brought the C:T ratio to equivalence (50%:50%). The current high frequency of C/C in subjects with spontaneous clearance of the HCV is compatible with the protective role of the C/C genotype against viral persistence. Recently, Fabris et al. [25] reported that in patients with chronic HCV infections, carriage of the T/T genotype occurred more frequently in those patients affected by ESLD than in those with mild chronic hepatitis, thus supporting the results of the present study. The present data also supports important recent results demonstrating that IL-28B polymorphisms are linked to a better response to antiviral treatment in patients with HCV chronic hepatitis [21][26][27] and to a higher probability of clearing the virus during the natural history of a chronic HCV infection [7][8]. In logical terms, the C/C genotype seems to be under-represented among patients with ESLD since carriage of the rs12979860 C/C genotype protects from unfavorable outcomes in chronic viral hepatitis C. In conclusion; in IL-28B rs12979860 C/T polymorphism the T allele appears to be more prevalent in patients with ESLD (LC and HCC). Besides, the C/C genotype is protective against the development of chronic HCV infection as well as later at the final stages of the disease, either directly or via its role in inhibiting HCV replication leading in most instances to spontaneous or IFN induced viral clearance during the early stages of infection.

## References

[1] Alter HJ, Seeff LB (2000). Recovery, persistence, and sequelae in hepatitis C virus infection: a perspective on long-term outcome.. Semin Liver Dis.

[2] Rehermann B (2009). Hepatitis C virus versus innate and adaptive immune responses: a tale of coevolution and coexistence.. J Clin Invest.

[3] Nash KL, Woodall T, Brown AS, Davies SE, Alexander GJ (2010). Hepatocellular carcinoma in patients with chronic hepatitis C virus infection without cirrhosis.. World J Gastroenterol.

[4] Morgan TR (2011). Chemoprevention of hepatocellular carcinoma in chronic hepatitis C.. Recent Results Cancer Res.

[5] Zekri AR, Moharram RA, Mohamed WS, Bahnassy AA, Alam El-Din HM, Abo-Shadi MM, Zayed NA, El-Magzangy H, Abdel-Aziz AO, Esmat G (2010). Disease progression from chronic hepatitis C to cirrhosis and hepatocellular carcinoma is associated with repression of interferon regulatory factor-1.. Eur J Gastroenterol Hepatol.

[6] Dring MM, Morrison MH, McSharry BP, Guinan KJ, Hagan R, O'Farrelly C (2011). Innate immune genes synergize to predict increased risk of chronic disease in hepatitis C virus infection.. Proc Natl Acad Sci U S A.

[7] Rauch A, Kutalik Z, Descombes P, Cai T, Di Iulio J, Mueller T, Bochud M, Battegay M, Bernasconi E, Borovicka J, Colombo S, Cerny A, Dufour JF, Furrer H, Günthard HF, Heim M, Hirschel B, Malinverni R, Moradpour D, Müllhaupt B, Witteck A, Beckmann JS, Berg T, Bergmann S, Negro F, Telenti A, Swiss Hepatitis C Cohort Study; Swiss HIV Cohort Study. (2010). Genetic variation in IL28B is associated with chronic hepatitis C and treatment failure: a genome-wide association study.. Gastroenterology.

[8] Thomas DL, Thio CL, Martin MP, Qi Y, Ge D, O'Huigin C, Kidd J, Kidd K, Khakoo SI, Alexander G, Goedert JJ, Kirk GD, Donfield SM, Rosen HR, Tobler LH, Busch MP, McHutchison JG, Goldstein DB, Carrington M (2009). Genetic variation in IL28B and spontaneous clearance of hepatitis C virus.. Nature.

[9] Fonseca-Coronado S, Vaughan G, Cruz-Rivera MY, Carpio-Pedroza JC, Ruiz-Tovar K, Ruiz-Pacheco JA, Escobar-Gutiérrez A (2011). Interleukin-28B genotyping by melt-mismatch amplification mutation assay PCR analysis using single nucleotide polymorphisms rs12979860 and rs8099917, a useful tool for prediction of therapy response in hepatitis C patients.. J Clin Microbiol.

[10] Lagging M, Askarieh G, Negro F, Bibert S, Soderholm J, Westin J, Lindh M, Romero A, Missale G, Ferrari C, Neumann AU, Pawlotsky JM, Haagmans BL, Zeuzem S, Bochud PY, Hellstrand K, DITTO-HCV Study Group. (2011). Response prediction in chronic hepatitis C by assessment of IP-10 and IL28B-related single nucleotide polymorphisms.. PLoS One.

[11] Chevaliez S, Hezode C (2010). IL28B polymorphisms and chronic hepatitis C.. Gastroenterol Clin Biol.

[12] Pineda JA, Caruz A, Di Lello FA, Camacho A, Mesa P, Neukam K, Rivero-juárez A, Macías J, Gómez-Mateos J, Rivero A (2011). Low-density lipoprotein receptor genotyping enhances the predictive value of IL28B genotype in HIV/hepatitis C virus-coinfected patients.. AIDS.

[13] Mihm S (2010). Hepatitis C virus, diabetes and steatosis: clinical evidence in favor of a linkage and role of genotypes.. Dig Dis.

[14] Petta S, Di Marco V, Di Stefano R, Cabibi D, Camma C, Marchesini G, Craxì A (2011). TyG index, HOMA score and viral load in patients with chronic hepatitis C due to genotype 1.. J Viral Hepat.

[15] Shah SR, Patel K, Marcellin P, Foster GR, Manns M, Kottilil S, Healey L, Pulkstenis E, Subramanian GM, McHutchison JG, Sulkowski MS, Zeuzem S, Nelson DR (2011). Steatosis is an independent predictor of relapse following rapid virologic response in patients with HCV genotype 3.. Clin Gastroenterol Hepatol.

[16] Cai T, Dufour JF, Muellhaupt B, Gerlach T, Heim M, Moradpour D, Cerny A, Malinverni R, Kaddai V, Bochud M, Negro F, Bochud PY, Swiss Hepatitis C Cohort Study Group. (2011). Viral genotype-specific role of PNPLA3, PPARG, MTTP, and IL28B in hepatitis C virus-associated steatosis.. J Hepatol.

[17] Youssef A, Yano Y, Utsumi T, abd El-alah EM, abd El-Hameed Ael E, Serwah Ael H, Hayashi Y (2009). Molecular epidemiological study of hepatitis viruses in Ismailia, Egypt.. Intervirology.

[18] Elkady A, Tanaka Y, Kurbanov F, Sugauchi F, Sugiyama M, Khan A, Sayed D, Moustafa G, Abdel-Hameed AR, Mizokami M (2009). Genetic variability of hepatitis C virus in South Egypt and its possible clinical implication.. J Med Virol.

[19] Bader el-Din NG, Abd el-Meguid M, Tabll AA, Anany MA, Esmat G, Zayed N, Helmy A, el-Zayady AR, Barakat A, el-Awady MK (2011). Human cytomegalovirus infection inhibits response of chronic hepatitis-C-virus-infected patients to interferon-based therapy.. J Gastroenterol Hepatol.

[20] Manolio TA (2010). Genomewide association studies and assessment of the risk of disease.. N Engl J Med.

[21] Ge D, Fellay J, Thompson AJ, Simon JS, Shianna KV, Urban TJ, Heinzen EL, Qiu P, Bertelsen AH, Muir AJ, Sulkowski M, McHutchison JG, Goldstein DB (2009). Genetic variation in IL28B predicts hepatitis C treatment-induced viral clearance.. Nature.

[22] Kelly C, Klenerman P, Barnes E (2011). Interferon lambdas: the next cytokine storm.. Gut.

[23] Liao XW, Ling Y, Li XH, Han Y, Zhang SY, Gu LL, Yu DM, Yao BL, Zhang DH, Jin GD, Lu ZM, Gong QM, Zhang XX (2011). Association of genetic variation in IL28B with hepatitis C treatment-induced viral clearance in the Chinese Han population.. Antivir Ther.

[24] Ruiz-Extremera A, Munoz-Gamez JA, Salmeron-Ruiz MA, de Rueda PM, Quiles-Perez R, Gila-Medina A, Casado J, Belén Martín A, Sanjuan-Nuñez L, Carazo A, Pavón EJ, Ocete-Hita E, León J, Salmerón J (2011). Genetic variation in interleukin 28B with respect to vertical transmission of hepatitis C virus and spontaneous clearance in HCV-infected children.. Hepatology.

[25] Fabris C, Falleti E, Cussigh A, Bitetto D, Fontanini E, Bignulin S, Cmet S, Fornasiere E, Fumolo E, Fangazio S, Cerutti A, Minisini R, Pirisi M, Toniutto P (2011). IL-28B rs12979860 C/T allele distribution in patients with liver cirrhosis: role in the course of chronic viral hepatitis and the development of HCC.. J Hepatol.

[26] Suppiah V, Moldovan M, Ahlenstiel G, Berg T, Weltman M, Abate ML, Bassendine M, Spengler U, Dore GJ, Powell E, Riordan S, Sheridan D, Smedile A, Fragomeli V, Müller T, Bahlo M, Stewart GJ, Booth DR, George J (2009). IL28B is associated with response to chronic hepatitis C interferon-alpha and ribavirin therapy.. Nat Genet.

[27] McCarthy JJ, Li JH, Thompson A, Suchindran S, Lao XQ, Patel K, Tillmann HL, Muir AJ, McHutchison JG (2010). Replicated association between an IL28B gene variant and a sustained response to pegylated interferon and ribavirin.. Gastroenterology.

